# A common origin of complex life cycles in parasitic flatworms: evidence from the complete mitochondrial genome of *Microcotyle sebastis *(Monogenea: Platyhelminthes)

**DOI:** 10.1186/1471-2148-7-11

**Published:** 2007-02-02

**Authors:** Joong-Ki Park, Kyu-Heon Kim, Seokha Kang, Won Kim, Keeseon S Eom, DTJ Littlewood

**Affiliations:** 1 Department of Parasitology, College of Medicine, Chungbuk National University, Cheongju, Chungbuk 361-763, Republic of Korea; 2 Korea Food and Drug Administration, Seoul 122-704, Republic of Korea; 3 School of Biological Sciences, Seoul National University, Seoul 151-747, Republic of Korea; 4 Department of Zoology, Natural History Museum, Cromwell Road, London SW7 5BD, UK

## Abstract

**Background:**

The parasitic Platyhelminthes (Neodermata) contains three parasitic groups of flatworms, each having a unique morphology, and life style: Monogenea (primarily ectoparasitic), Trematoda (endoparasitic flukes), and Cestoda (endoparasitic tapeworms). The evolutionary origin of complex life cyles (multiple obligate hosts, as found in Trematoda and Cestoda) and of endo-/ecto-parasitism in these groups is still under debate and these questions can be resolved, only if the phylogenetic position of the Monogenea within the Neodermata clade is correctly estimated.

**Results:**

To test the interrelationships of the major parasitic flatworm groups, we estimated the phylogeny of the Neodermata using complete available mitochondrial genome sequences and a newly characterized sequence of a polyopisthocotylean monogenean *Microcotyle sebastis*. Comparisons of inferred amino acid sequences and gene arrangement patterns with other published flatworm mtDNAs indicate Monogenea are sister group to a clade of Trematoda+Cestoda.

**Conclusion:**

Results confirm that vertebrates were the first host for stem group neodermatans and that the addition of a second, invertebrate, host was a single event occurring in the Trematoda+Cestoda lineage. In other words, the move from direct life cycles with one host to complex life cycles with multiple hosts was a single evolutionary event. In association with the evolution of life cycle patterns, our result supports the hypothesis that the most recent common ancestor of the Neodermata giving rise to the Monogenea adopted vertebrate ectoparasitism as its initial life cycle pattern and that the intermediate hosts of the Trematoda (molluscs) and Cestoda (crustaceans) were subsequently added into the endoparasitic life cycles of the Trematoda+Cestoda clade after the common ancestor of these branched off from the monogenean lineage. Complex life cycles, involving one or more intermediate hosts, arose through the addition of intermediate hosts and not the addition of a vertebrate definitive host. Additional evidence is required from monopisthocotylean monogeneans in order to confirm the monophyly of the group.

## Background

The evolutionary origin of parasitism throughout the tree of life remains a central issue in evolutionary biology and has attracted intense theoretical and empirical laboratory-based attention. In this respect the Platyhelminthes ('flatworms') have received great attention from evolutionary biologists as a model system for investigating the adaptive radiation associated with the evolution of parasitism (e.g. see [1]). The phylum is represented by an assemblage of superficially simple metazoan animal groups and has long been considered to provide a key to understanding the evolutionary origin and diversification of bilaterally symmetrical metazoan groups [2]. It includes about 100,000 extant species of both free-living and parasitic forms [3]. The conventional view of the phylum 'Platyhelminthes' is that it contains three major clades Acoelomorpha (Acoela+Nemertodermatida), Catenulida, and Rhabditophora [4], but recent phylogenetic studies based on morphological [5] and molecular evidence [6-10] have suggested non-monophyly (mostly polyphyly) of the Platyhelminthes. This is inconsistent with long-held prevailing concept of "the phylum Platyhelminthes" as defined in most zoological textbooks. Subsequent studies have separated the Acoelomorpha from the remaining catenulid and rhabditophoran Platyhelminthes, with the acoelomorphs occupying a pivotal basal position among the Bilateria [11,12] and the Platyhelminthes (*sensu stricto*) as relatively derived members of the Lophotrochozoa [13]. Nevertheless, it is generally accepted that the Platyhelminthes contains four major groups, each having a unique anatomy, body size, and life style: the 'Turbellaria' (a paraphyletic assemblage of at least seven distinct lineages of mostly free-living forms), Monogenea (primarily ectoparasitic), Trematoda (endoparasitic flukes), and Cestoda (endoparasitic tapeworms). Among these, the latter three groups (called 'Neodermata'; [14]) are represented by diverse obligate parasitic flatworms of invertebrates and vertebrates that cause diseases in a variety of host animal groups, including domestic animals and humans.

**Figure 1:**
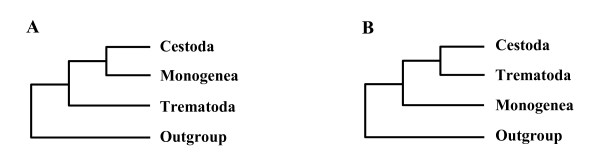
Summaries of the previous phylogenetic hypotheses of relationships among three major neodermatan groups. (A) Morphology [1,34,35,38]; 18S rDNA [17,36]; Morphology+18S rDNA [7,43]. (B) 28S rDNA [20]; 18S+28S rDNA [19].

The monophyletic grouping of neodermatans is considered to be beyond doubt [15,16], but sister-group relationships among its subordinate groups (i.e., Monogenea, Trematoda, and Cestoda) are still under vigorous debate [3,17-19]. Depending on the data sources employed for phylogenetic analysis by previous authors, relationships inferred were not compatible with each other (see Fig. 1 for details). Although a closer relationship of Monogenea with Cestoda has received broader support from morphological and molecular sequence data, phylogenetic analyses of partial 28S rDNA sequences [20] and complete 28S plus complete 18S sequences [19] suggested a different result, rejecting a long established clade of Cestoda+Monogenea (= Cercomeromorphae). Inconsistency in phylogenetic conclusions by previous authors concerning sister-group relationships among three major neodermatan groups, suggests the need for additional independent molecular markers to resolve this issue.

The Monogenea are composed of mostly ectoparasitic species which live on external organs (e.g., gill, skin, etc.) of a broad range of aquatic vertebrate host, especially fishes, with some exceptional species occurring in internal organs of the host [21]. As illustrated in Fig. 1, the phylogenetic position of the Monogenea, and its monophyly, remain outstanding issues, because the evolutionary origin of ecto- and endo-parasitism in major parasitic platyhelminth groups can be elucidated only if its phylogenetic position within the Neodermata clade is fully resolved [22]. If Monogenea are resolved as paraphyletic, occupying the earliest branching lineages, then ectoparasitism as the earliest life habit of neodermatans may be inferred with confidence.

With a few exceptions, animal mitochondrial genomes are circular in form, ranging from 13~16 kb in size. Each genome contains 37 genes: 13 protein-coding genes, two ribosomal RNA genes and 22 tRNA genes [23]. Comparative analysis of the mitochondrial genome information (e.g., gene arrangement, nucleotide and amino acid sequences) has become a popular molecular tool for resolving the deeper node of phylogenetic interrelationship in a variety of metazoan groups [24-27]. To date, the complete mitochondrial genome sequence has been determined for 13 flatworm species (six from cestodes and seven from digenean trematodes), but the taxon sampling is highly biased toward endoparasitic flatworms i.e. cestodes and trematodes that are of medical or economic importance (for details see [28]). No complete mitochondrial genome information from monogenean species has been available as yet and this lack of information has hindered better understanding of the impending phylogenetic issue as well as mitochondrial genome evolution among major lineages of the parasitic Platyhelminthes. For these reasons, comparisons of a monogenean mitochondrial genome with other flatworms are expected to be very useful for resolution of phylogenetic relationships in conjunction with gene rearrangement among the mitochondrial genomes of three neodermatan groups (Cestoda, Trematoda, and Monogenea). In the present study, we revisited the phylogenetic issue of the Monogenea within the parasitic Platyhelminthes based on the comparisons of the complete mitochondrial genome sequences of a polyopisthocotylid monogenean,*Microcotyle sebastis *Goto, 1894 with published data from other flatworm species.

## Results and Discussion

### General Features of *M. sebastis *mtDNA Genome

The complete mitochondrial genome of *M. sebastis *[GenBank accession number: DQ412044] is 14,407 bp in size, one of the largest flatworm mitochondrial genomes published to date. The genome is composed of 36 genes (all genes are encoded in the same direction) consisting of 12 protein-coding genes, 22 tRNA genes and two ribosomal RNA genes but lacking the *atp8 *gene, which is a common feature in flatworm mtDNAs (see Additional File 1). *M. sebastis *mitochondrial DNA (mtDNA) is A+T-rich (A+T content of 70.5%; 29.4% A, 41.1% T, 19.8% G, and 9.7% C) (Table 1). The A+T-richness of the entire sequence is more or less similar to those in some cestodes (71.0% of A+T in *H. diminuta*; [29]) and schistosomes (71.0%, 72.2% in *S. japonicum *and *S. mekongi*, respectively; [30]), whereas this value is considerably higher than that found in *F. hepatica *(63.5%) and *P. westermani *(51.5%) (see [30] for details). The values for AT-skew and GC-skew of *M. sebastis *mtDNA were calculated according to the formulae ([A-T]/[A+T]) for the former, ([G-C]/[G+C]) for the latter [31]. For the entire sequences, AT skew and GC skew are -0.17, 0.34, respectively. The skewness in protein coding sequences (AT skew = -0.20, GC skew = 0.34) is very similar to that of entire sequences, but the AT skewness is relatively lower for the tRNA genes, ribosomal RNA genes and non-coding region (Table 1). The lower value of AT skewness in these genes is generally considered to be associated with the formation of stem-loop secondary structures (i.e., base pairing between A and T in the stem regions of their corresponding secondary structures). It is notable that the extremely high value of GC skewness (0.85) was encountered in the highly repetitive region (see below for details). This unexpectedly high GC skewness is due to the configuration of a stem-loop structure with some cases of G:T base pairing (instead of G:C) in each of nine 53nt-repeat units.

**Table 1:** Nucleotide composition and AT- and GC-skewnesses of *M. sebastis *mtDNA sequences for potein-coding, rRNA, tRNA genes and non-coding regions.

Nucleotide		Length (bp)	A (%)	C (%)	T (%)	G (%)	A+T (%)	G+C (%)	AT-skew	GC-skew
Entire sequence		14,407	29.4	9.7	41.1	19.8	70.5	29.5	-0.17	0.34
Protein-coding sequence^a^		10,254	27.8	10.1	41.8	20.3	69.6	30.4	-0.20	0.34
Codon position	1st	3,418	29.6	9.6	37.3	23.5	66.9	33.1	-0.12	0.42
	2nd	3,418	19.8	13.0	46.1	21.1	65.9	34.1	-0.40	0.24
	3rd	3,418	34.0	7.5	42.1	16.4	76.1	23.9	-0.10	0.37
Ribosomal RNA gene sequence		1,696	33.1	10.7	38.1	18.1	71.2	28.8	-0.07	0.26
Transfer RNA gene sequence		1,424	32.6	9.8	38.8	18.8	71.4	28.6	-0.09	0.31
Highly repetitive region (HRR)		472	33.9	1.9	41.5	22.7	75.4	24.6	-0.10	0.85
Unassigned region (UAR)		348	33.7	8.6	44.5	13.2	78.2	21.8	-0.14	0.21

^a^Termination codons were not included.

The majority of protein-coding genes (ten of 12 genes; *cox2, cox3, cob, atp6, nad1, nad2, nad3, nad4L, nad5 *and *nad6*) appear to use ATG as the start codon, while the other genes are predicted to start with ATT (*cox1*) and ATA (*nad4*), respectively. Seven of the 12 genes terminate with TAA (*cox3, nad4, atp6, nad2, nad3, cox1 *and *nad6*) and the other three use TAG (*cob, nad4L *and *cox2*) as the termination codon. Although incomplete termination is common in metazoan mtDNAs [23,24], it is relatively rare in the flatworms studied thus far. Only two genes (*nad5 *and *nad1*) are inferred to end with incomplete codon TA and T, respectively, each of these is immediately adjacent to the downstream tRNA genes *trnE *and *trnN*.

Twenty-two nucleotide sequence segments (ranging in size from 59 nt [*trnS1*] to 69 nt [*trnL1*]) were predicted to fold into a cloverleaf secondary structure (see Additional File 2). The putative secondary structures common in 22 tRNA genes include an amino-acyl stem of 7 nucleotide pairs (ntp), a DHU-stem of 3-4 ntp with a 4-9 nt loop, an anticodon stem of 5 ntp with a loop of 7 nt, and a TΨC stem of 3-6 ntp with a loop of 3-6 nt. Some exceptions to these common features are *trnS1 *(AGN) and *trnS2 *(UCN) in which each of DHU arms is missing and replaced with an unpaired loop (10-14 nt) as found in all other flatworm species [32]. Anticodon sequences of 22 tRNAs were identical to each of their corresponding tRNA genes found in other flatworm species with an exception that the *trnR *has a TCG anticodon sequence, rather than ACG as those found in other platyhelminth groups.

A total of 21 intergenic regions, varying from a single nucleotide to 472 nt long in size, were found in the *M. sebastis *mtDNA genome. Of these, two intergenic sequences, i.e., the highly repetitive region (HRR; 472 nt) and unassigned region (UAR; 348 nt) adjacent to each other are most prominent. The HRR located between *trnK *and UAR contains seven identical repeat units of a 53-nt sequences plus two additional repeat units, each abutting directly onto upstream and downstream of seven consecutive repeat units, respectively, with some sequence modifications: two substitutions (T→G and A→T substitutions at the second and fifth positions of 53-nt repeat unit; upstream unit) or with a truncated sequence of 5-nt from 3' end of the repeat unit (downstream unit). A 53-nt repeat unit is predicted to form a stem-loop secondary structure (not shown) with a 21 base paired-long stem and a loop of 9 nucleotides. Of 21 base pairs in the stem region, there are 12 A:T, five G:T, one G:C, three mismatch pairings (1 G:G and 2 A:G) and two unpaired A's, respectively. The highly structured A:T and G:T base pairings with avoiding C (only a single C is detected in each of the repeated unit) account for an extremely high level of GC skewness (0.85) in this repeat region. Although there are some mismatched base pairings and two unpaired A's in the stem region, the predicted putative secondary structure is considered to be analogous to those reported in cestode species (*H. diminuta *[29]; *T. asiatica *[33]). This stem-loop structure, although its function is still unclear, has often been assumed to be associated with replication origin. The second largest intergenic sequence, an unassigned region (UAR), is located between the HHR and *nad6*. This region contains a peculiar ORF (open reading frame)-like sequence segment of 174 nt comprising 58 codons including the starting codon (GTG for Val) and termination codon TAA. Although *atp8 *gene has never been found in flatworm mitochondrial genomes, the size of the unattributed ORF is more or less similar to the *atp8 *genes reported in other metazoans. In order to confirm its identity, we conducted the hydropathic profile comparison with other published *atp8 *genes (*Geodia neptuni *[Porifera]; *Paratomella rubra *[Acoelomorpha]; *Trichinella spiralis *[Nematoda]; *Lumbricus terrestris *[Annelida]; *Limulus polyphemus *[Arthropoda]; and *Homo sapiens *[Vertebrata]) using MacVector program (Accelrys Inc.). The predicted hydropathy profiles of the ORF candidate displayed very different patterns from the other sequences compared. Direct sequence comparisons of the ORF candidate using both nucleotide and amino acid sequence were also performed, but nearly no similarity was detected. Moreover, the AT content of the ORF candidate (78.3%) was much higher than the average (69.6%) of 12 protein-coding genes. Thus, taken together the evidence does not provide unambiguous support that this sequence is *atp8 *and therefore we designated it as an unassigned region (UAR).

### Molecular Phylogeny of the Neodermata

**Figure 2:**
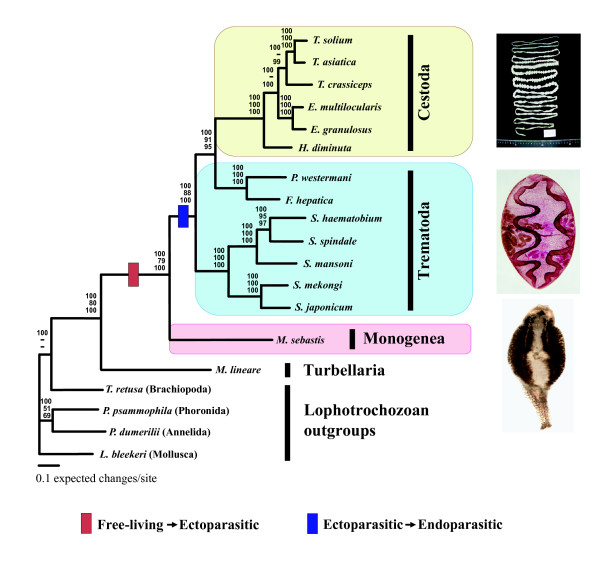
Phylogenetic relationships among the neodermatan groups based on the analysis of amino acid sequence data from 12 mitochondrial protein-coding gene loci using the Bayesian analysis, maximum likelihood, and maximum parsimony methods. Numbers above the branches represent Bayesian posterior probabilities, quartet puzzling supporting values, and parsimony bootstrap values, respectively. The branches that were not universally supported with values of ≤ 50% are represented by "-" in each supporting values of the node.

As explained, we prepared three independent amino acid sequence datasets for phylogenetic analyses. From 12 protein-coding genes (using dataset #1, containing all complete neodermatan sequences and the incomplete *M. lineare *sequences as ingroups, and four complete lophotrochozoan sequences as outgroups), a concatenated amino acid sequence dataset containing 2,506 homologous positions was prepared for the following phylogenetic analyses. Implementation of the maximum likelihood mapping analysis showed that more than 97.9% of all random samples of the quartet (33.0%, 31.7% and 33.0 in each trapezoid; data not shown) were fully resolved, indicating that the dataset contains a high level of tree-like phylogenetic information. Of 2,506 homologous positions, 1,901 variable sites were phylogenetically informative under the maximum parsimony (MP) criterion. Phylogenetic relationships among major neodermatan groups using Bayesian inference (BI), maximum likelihood (ML), and maximum parsimony (MP) analyses are shown in Fig. 2. The heuristic search option of the maximum parsimony criterion using inferred gaps as missing characters generated a single most-parsimonious tree (tree length = 11,373 steps, CI = 0.722, RI = 0.674) as represented in Fig. 2. All three phylogenetic methods produced the same tree topology in the branching patterns except for one minor change in their respective position between *T. crassiceps *and *E. multilocularis*: The BI and MP found *Echinococcus *species (*E. multilocularis *and *E. granulosus*) sister to three *Taenia *species, whereas the ML analysis showed relationships of ((*T. solium*, *T. asiatica*) (*E. multilocularis, E. granulosus*)) *T. crassiceps*). In all phylogenetic methods used *M. sebastis*, representing the Monogenea, was positioned as sister to the Trematoda+Cestoda clade comprising all endoparasitic members. Phylogenetic analyses of dataset #2 (containing the complete neodermatan sequences only [excluding the incomplete *M. lineare *sequences] as ingroups, and four complete lophotrochozoan sequences as outgroups), and dataset #3 (comprising five gene loci only [*nad5, cox3*, *atp6, cox1 *and *cob*] obtained universally from all platyhelminth ingroup and lophotrochozoan outgroup taxa) yielded trees identical to that shown in Fig. 2. This result is not concordant with the long-standing prevailing hypothesis that has favored closer relationship between the Monogenea and Cestoda based on morphology [34,35] and 18S rRNA molecules [17,36]. The concept of 'Cercomeromorphae' (an assertion that Monogenea are more closely related to Cestoda than to Trematoda) was first advocated by Janicki [37]. Brooks and his colleagues [1,34,38] have persistently advocated strong support for the 'Cercomeromorphae' clade (Monogenea+Cestoda) based on phylogenetic surveys of morphological characters. With respect to the possession of a hook-bearing posterior end structure in larval stages of tapeworms (Cestoda), considered as being homologous to the posterior opisthaptor of monogenean groups, it has long been accepted from morphological systematists as a key character uniting Cestoda and Monogenea [34,35]. Furthermore, as shown in Fig. 1, many recent works based either on molecular data, or on the combined dataset of morphology+molecular sequence have suggested different conclusions concerning the sister-relationships among three neodermatan groups, depending on the data sources examined. Of these, our data are consistent with that of the most recently published result of molecular phylogenetic analysis of the combined complete 18S+28S rDNA data [19] in which the conventional concept of 'Cercomeromorphae' was not supported. The position of *M. sebastis *was strongly supported by high bootstrap resampling and posterior probability values (100%) in the analyses of MP and BI, but received slightly lower support (88%) in the ML analysis. In order to evaluate the stability of the monogenean position in the Neodermata clade, the unconstrained 'best tree' was statistically tested with each of the alternative phylogenetic hypotheses (constrained trees) using the parsimony-based Templeton test [39] and likelihood-based Shimodaira-Hasegawa test (SH test; [40]) implemented in PAUP* 4.0b10 and TREE-PUZZLE 5.2, respectively. Resulting tree statistics demonstrate a significant difference (Table 2) between the unconstrained optimal topology (a close affinity of Trematoda with Cestoda) and the constrained alternative topology (sister-group relationship of Monogenea/Cestoda), indicating that the position of Monogenea within the Neodermata clade is robustly corroborated by the current mtDNA dataset. This is the first mitochondrial genome-based molecular phylogeny supporting the position of the Monogenea as sister group to the Trematoda+Cestoda. Consequently, our result adds another independent line of molecular evidence that refutes the 'Cercomeromorphae' hypothesis. This interpretation is further supported by the comparison of gene arrangement patterns of the flatworm mitochondrial genomes. In order to statistically evaluate the paraphyly of the Trematoda found in all analyses, the Templeton test (MP) and Shimodaira-Hasegawa test (ML) were applied to unconstrained 'best tree' and constrained (monophyletic Trematoda) phylogenetic estimates. Although MP based phylogenies were not significantly different, those estimated using ML were (Table 2). There is no evidence to suggest that Trematoda, or indeed the Digenea or those digenean taxa included, are not monophyletic from nuclear genes or morphology. We suspect that resolving the Trematoda as paraphyletic is likely artefactual, but we can only speculate as to what may have caused this bias; e.g. base compositional differences between schistosomes and other digeneans (particularly *Paragonimus*), poor taxon sampling (all the cestodes are highly derived cyclophyllideans). Additional sequences from a broad taxon sampling of both cestodes and trematodes and gene-by-gene analyses may resolve this anomaly.

**Table 2:** The statistics of tree topology test using Shimodaira-Hasegawa [40] and Templeton [39] tests for comparisons of alternative hypothesis.

Phylogenetic hypotheses	Tree length	Length difference	-ln*L*	Difference in -ln*L*	*P*-value
Unconstrained best tree			57675.67		
Constrained tree					
Monophyly of Cestoda+Monogenea			67211.72	9536.04	0.0000
Monophyly of Trematoda			67350.49	9674.82	0.0000
Unconstrained MP tree	11,373				
Constrained tree					
Monophyly of Cestoda+Monogenea	11,427	54			0.0009
Monophyly of Trematoda	11,395	22			0.1451

**Figure 3:**
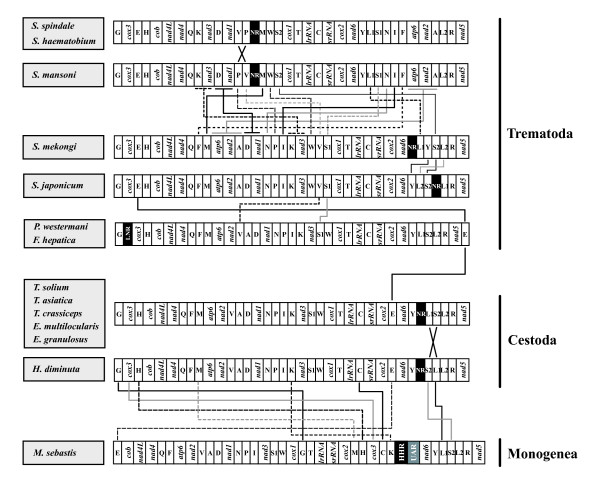
Linearized comparison of the mitochondrial gene arrangement of 13 flatworm species. Gene and genome size are not to scale. All genes are transcribed in the same direction (from left to right). The tRNAs are labeled by single-letter abbreviations and two leucine and two serine tRNA genes are marked, according to their anticodon sequence, as L1 (*trnL-uag*), L2 (*trnL-uaa*), S1 (*trnS-gcu*), and S2 (*trnS-uga*), respectively. The non-coding regions are denoted by the NR according to the previous authors. An unassigned region, located between the HHR (highly repetitive region) and *nad6 *in *M. sebastis *mtDNA is marked by UAR. Homologous genes between the taxa are indicated by connected lines.

The gene arrangement of *M. sebastis *displays notably different patterns, compared to those found from other flatworm species (Fig. 3). The difference in the gene arrangement of *M. sebastis *from other flatworms includes some translocations of the protein-coding genes (*cox3 *and *nad6*), tRNA genes (*trnE*, *trnG*, *trnM*, *trnH*, *trnC*, and *trnK*), and two noncoding regions (highly repetitive region and unassigned region called as HRR and UAR, respectively). In general, the flatworm mtDNA gene order appears relatively well conserved, with the exception of the African *Schistosoma mansoni *and other schistosomes *S. haematobium *and *S. spindale *whose gene order is markedly different from those of other flatworms including other congeneric East Asian lineages *S. japonicum*, *S. mekongi*, and *S. malayensis *[30]. A major gene rearrangement among these schistosomes was interpreted as being supporting evidence for phyletic differentiation between the two independent geographic clades [41]. Aside from those of schistosomes, all gene arrangement changes encountered between cestode and trematode groups are remarkably minor and limited to very few translocations among some tRNA genes (*trnE*, *trnV*, *trnW*, *trnL1*, and *trnS2*) and the non-coding regions. This indicates that cestodes and trematodes show high similarity in their gene arrangement pattern, but differ substantially from the monogenean member *M. sebastis*. The shared gene arrangement among cestodes and major lineages of trematode members is very likely to reflect a common ancestry, rather than being due to convergent evolutionary events. Along with a robust sister-group relationship in all phylogenetic analyses of amino acid sequences, gene arrangement data also provide the supporting evidence for the closer relationship between Cestoda and Trematoda. Nevertheless, we are still limited by the lack of information from the closest outgroup of the Neodermata with which to detect the plesiomorphic condition within the Neodermata. Recently, the partial sequences of the mitochondrial genome became available (approximately 6.8 kb) for a macrostomid 'turbellarian' *Microstomum lineare *[9], but this showed almost no shared gene boundaries with the neodermatan groups, making it intractable for use in phylogenetic analysis using gene arrangement. Further comparisons of gene arrangements based on more sampling of the complete mitochondrial genomes from the closest outgroup taxa will provide useful information for resolving the issue of mitochondrial genome evolution among major groups of the flatworms.

### Evolution of Parasitism in the Neodermata

Evolutionary diversification and adaptive radiation of the parasites are strongly correlated with their life cycle patterns. Accordingly, the evolutionary history of major parasitic platyhelminth groups can be traced by examining the evolutionary changes of parasite-host associations [42]. Tracking back to their historical transformations of different life history forms during the evolutionary process can convincingly be clarified only if it is interpreted within a robust phylogenetic framework. The interrelationships of the major neodermatan lineages is required to determine how parasitic platyhelminths evolved, and which life cycle pattern (ectoparasitism vs. endoparasitism) arose first within the Neodermata clade. If monogenean monophyly is true, as supported by the majority of previous works, it is expected that a single shifting event from ecto- to endo-parasitism or vice versa is equally possible to explain the evolutionary scenario of the parasitic patterns found in the neodermatan groups. An endoparasitic life style appears to be plesiomorphic when inferred from morphological [1,34,35,38] and molecular [7,43] phylogenies where Trematoda are sister group to Cestoda+Monogenea. In contrast, in gene trees where Cestoda and Trematoda are sister taxa [19,20] (Fig. 1B), it can be postulated that ectoparasitism is the plesiomorphic condition. Indeed, it has often been considered among parasitologists that a parasitic group with a simple (direct) life cycle and higher specificity to its host groups is more primitive than the group with more complicated life cycles [15,44]. Therefore, the simple, direct life cycle pattern displayed by monogenean groups has been assumed to be primitive and also correlated with high level of host specificity [1]: Trematodes and cestodes display rather complicated life cycle, such as utilizing many invertebrates i.e., molluscs (trematodes) or arthropods (cestodes) as intermediate hosts prior to entering into the final life stage in various vertebrate hosts ('multiple host system'). In contrast, the majority of Monogenea are ectoparasitic on a broad range of aquatic vertebrates and have only one host with high host specificity during their life cycle, not involving intermediate hosts ('single-host system'). The position of Monogenea in the mitochondrial gene tree (Fig. 2) is compatible with the idea that a parasitic group maintaining simpler life cycle (being ectoparasitic) is more primitive than the group with more complicated life cycles (being endoparasitic), if we accept that a single host life cycle precedes a two or more host life cycle. Considering it in conjunction with the interpretation of life cycle patterns within the Neodermata, our mitochondrial gene tree corroborates the idea that ectoparasitism arose first in the neodermatan phylogeny (i.e. primitive condition), probably in an early lineage of monogenean groups, and that endoparasitism was secondarily acquired (i.e. derived condition) in cestodes and trematodes (comprising all extant obligate endoparasitic forms) after their common ancestry diverged from the Monogenea. This result contradicts the previous hypotheses that the common ancestor of the Neodermata acquired endoparasitism as the first mode of parasitism.

### Evolution of Host-Parasite Association in the Neodermata

**Figure 4:**
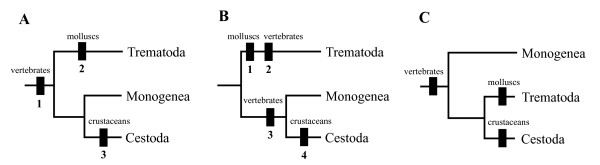
Different views in the interpretations of the host adoption within the neodermatan phylogenetic framework. The relative sequence in the acquisition of invertebrate/vertebrate hosts by three major groups of the Neodermata is illustrated by numerical order (not to scale). (A) 'Vertebrate first' hypothesis; (B) 'Mollusc first' hypothesis (modified [45]); (C) the interpretation of the present study.

During the last decades, there has been a debate regarding the order of host types in parasitic platyhelminths: what host type was the earliest form involved in the life cycles of the common ancestor of the Neodermata, i.e., 'Vertebrate first' vs. 'Mollusc first' (Fig. 4)? Under the parsimony assumption, in which the hypothesis with fewer inferences of character transformation is preferred, mapping of host adoption by each of the stem groups of the Neodermata onto the previously accepted phylogenetic relationships has suggested two different ways of interpretation in relation to their life cycle evolution [45]. The simplest, most parsimonious scenario ('Vertebrate first') proposed first by Littlewood et al [43] was that the proto-trematodes first acquired an endoparasitic association with vertebrates and that independent adoptions of invertebrates by the Trematoda (molluscs) and Cestoda (crustaceans) were subsequent acquisitions (Fig. 4A). On the other hand, it has also been suggested that the association of common ancestor of the Trematoda with molluscan hosts was primitive (acquiring its subsequent vertebrate hosts independently), and that the vertebrates were involved in the life cycle of the common ancestor of the Monogenea+Cestoda clade as independent initial hosts apart from that of trematodes, with the crustaceans as subsequent intermediate hosts adopted by the Cestoda groups after the ancestral cestode was diverged from the monogeneans ('Mollusc first'; Fig. 4B). The 'Mollusc first' hypothesis has received relatively broader support from the authors who intended to explain the digenean evolution in relation to host distribution [15,46]. This view was based on some assumptions that the Aspidogastrea is the most primitive group among the Neodermata [47], and that the Trematoda (Aspidogastrea+Digenea) is the sister group of all the other neodermatan groups. But this scenario is not parsimonious, because it necessitates extra assumptions of secondary loss and/or gain of invertebrate (molluscs and crustaceans) and vertebrate hosts during the divergence of major groups of the Neodermata. Notwithstanding the difference in their way of interpretation, both of these alternative explanations were made on the basis of previous phylogenetic assertion that Monogenea and Cestoda are most closely related ('Cercomeromorphae') and that the Trematoda is sister to the Monogenea+Cestoda. The underlying phylogenetic hypothesis on which most of previous authors relied is seriously challenged by the conclusion of the present study in which the Monogenea are positioned as sister group of the Trematoda+Cestoda, refuting the 'Cercomeromorphae' theory. A possible scenario, not refuted by our mitochondrial gene tree, is that the monogenean lineage is the earliest offshoot within the Neodermata clade. However, further verification is required that the Monogenea are indeed a monophyletic group; paraphyly, with Trematoda+Cestoda as the most derived lineage would confirm this scenario. If Monogenea were paraphyletic it would be most parsimonious to infer monogeneans had diverged before either trematodes or cestodes. If the interpretation of monogenean evolution suggested here is correct (Fig. 4C), then it is convincing to postulate that the most recent common ancestor of the Neodermata giving rise to the Monogenea adopted vertebrate ectoparasitism as its initial life cycle pattern and that the intermediate hosts of the Trematoda (molluscs) and Cestoda (crustaceans) were subsequently added into the endoparasitic life cycles of the Trematoda+Cestoda clade after the common ancestor of these branched off from the monogenean lineage. Vertebrate parasitism first (whether ecto- or endo-) is fully supported. Although some authors have argued that cestodes are the most derived of all Neodermata (see [15]), there is no conclusive evidence available for ordering which intermediate host form was first introduced into the life cycles of trematodes and cestodes. Our interpretation based on the mitochondrial genome phylogeny is likely to be the simplest (most parsimonious) explanation in understanding the evolution of host-parasite associations coupled with the modes of parasitism in the Neodermata. As Cribb et al [48] pointed it out in detail, however, evolutionary estimation of the character transformation using a parsimony approach does not always produce the best solution and it might be misleading when information utilized for the analysis is incomplete. The uncertainty of the closest relatives to the Neodermata and its life cycle pattern has not been resolved yet, and therefore our interpretation should be further verified by evidence concerning the identity of the closest sister group of the Neodermata and the life cycle mode in which it was engaged. Thereafter we will be much more confident of the conclusion that the ectoparasitism arose first within the Neodermata representing the vast majority of parasitic flatworms.

## Conclusion

The evolutionary origin of parasitism within the Neodermata was inferred for the first time from a phylogeny of the Neodermata, estimated with complete mitochondrial genomes from all parasite classes. Comparisons of inferred amino acid sequences and gene arrangement patterns of a polyopisthocotylid monogenean *Microcotyle sebastis *with other published flatworm mtDNAs indicate that Monogenea are sister group to the Trematoda and Cestoda within the Neodermata clade. From this finding, we suggest that ectoparasitism likely arose first in the neodermatan phylogeny (i.e. primitive condition), probably in an early lineage of monogenean groups, and that endoparasitism was acquired secondarily (i.e. derived condition) in cestodes and trematodes after their common ancestry diverged from the Monogenea. In association with the evolution of life cycle patterns, our result lends strong evidence that the most recent common ancestor of the Neodermata giving rise to the Monogenea adopted vertebrate ectoparasitism as its initial life cycle pattern and that the intermediate hosts of the Trematoda (molluscs) and Cestoda (crustaceans) were subsequently added into the endoparasitic life cycles of the Trematoda+Cestoda clade after the common ancestor of these branched off from the monogenean lineage. Complex life cycles, involving one or more intermediate hosts, arose through the addition of intermediate hosts and not the addition of a vertebrate definitive host.

## Methods

### Sampling and Molecular Techniques

Live specimens of *M. sebastis *were isolated from the gill of host fish *Sebastes schlegeli *from a fish farm at the Namhae County of Gyeongsangnam-do Province of South Korea (N 34°42'23", E 128°03'97"). The total genomic DNA was extracted from a single individual using a QIAamp tissue kit (Qiagen Co.). Two partial fragments of *cob *(~450 nt) and *rrnL *(~430 nt) were initially PCR-amplified and cycle-sequenced using two primer sets: The *cob *primers (Cytb-424F [5'-GGW TAY GTW YTW CCW TGR GGW CAR AT-3'] and Cytb-876R [5'-GCR TAW GCR AAW ARR AAR TAY CAY TCW GG-3']) were originally designed by von Nickisch-Rosenegk, Brown, and Boore [29] and the *rrnL *primers (PL16SF [5'-WYYGTGCDAAGGTAGCATAAT-3'] and PL16SR [5'-AWAGATAAGAACCRACCTGGCT-3']) were directly designed on the basis of conserved regions of mitochondrial 16S rDNA sequences of diverse platyhelminth species. The sequences obtained in these two regions were then used to design species-specific primer sets for long PCR reactions. Two pieces of overlapping long PCR products (~7.8 and ~7.4 kb each in size) covering the entire mitochondrial genome were amplified using the Expand Long Template PCR System (Roche Co.) with the following conditions: 1 cycle of initial denaturation (45s at 94°C), 35 cycles of denaturation-primer annealing-elongation (10s at 92°C, 30s at 63°C, and 8 min at 68°C), and 1 cycle of the final extension (12 min at 72°C). A negative control (no template) was also performed for every PCR run to determine any potential contamination of the PCR products. The amplified long PCR products were isolated on a 0.8% agarose gel containing crystal violet, excised in ambient light and extracted using the TOPO Gel Purification reagents supplied with the TOPO XL Cloning kit (Invitrogen Co.). After gel purification, each of two long PCR products was ligated using the cloning kit (TOPO XL Cloning kit) and then transformed into *E. coli *competent cells. Cyclic sequencing reactions for each of the long PCR products were performed in both directions with a Big Dye Terminator Cycle-Sequencing Kit (Applied Biosystems) using primer walking. A full strand of the entire mtDNA sequence was then assembled by double-checking the sequences of overlapping regions of the two long PCR fragments.

### Gene Annotation and Phylogenetic Analyses

Twelve protein-coding genes and two ribosomal RNA genes of *M. sebastis *were identified by sequence comparison with those published in other flatworm species, with the aid of a web-based automatic annotation program for organellar genomes (DOGMA; [49]). We identified the putative secondary structures of 22 tRNA genes by using tRNAscan-SE program [50] or by recognizing potential secondary structures and anticodon sequences by visual inspection. The amino acid sequences for protein-coding genes of *M. sebastis *mtDNA were inferred using the flatworm mitochondrial genetic code (the genetic code table 9 in GenBank). Amino acid sequences, and gene starts and stops, were verified by alignment against homologous genes from other flatworms. In addition to the mtDNA of *M. sebastis*, the mitochondrial genome sequences for 13 neodermatan species and four lophotrochozoan species (used as outgroups) were retrieved from the GenBank for phylogenetic analyses: *Echinococcus multilocularis *[GenBank: NC_000928], *E. granulosus *[GenBank: NC_008075], *Fasciola hepatica *[GenBank: NC_002546], *Hymenolepis diminuta *[GenBank: NC_002767], *Paragonimus westermani *[GenBank: NC_002354], *Schistosoma japonicum *[GenBank: NC_002544], *S. mansoni *[GenBank: NC_002545], *S. mekongi *[GenBank: NC_002529], *S. spindale *[GenBank: NC_008067], *S. haematobium *[GenBank: NC_008074], *Taenia asiatica *[GenBank: NC_004826], *T. crassiceps *[GenBank: NC_002547], *T. solium *[GenBank: NC_004022], *Loligo bleekeri *[GenBank: NC_002507; Mollusca], *Phoronis psammophila *[GenBank: AY368231; Phoronida], *Platynereis dumerilii *[GenBank: NC_000931; Annelida], and *Terebratulina retusa *[GenBank: NC_000941; Brachiopoda]. The sequence information of the mitochondrial protein-coding genes for rhabditophoran turbellarian species *Microstomum lineare *is limited to smaller number of gene loci (full lengths of *nad5, cox3*, *atp6 *and partial lengths of *cox1 *and *cob*; [9]). For this reason, three independent amino acid sequence datasets were prepared for phylogenetic analyses: (1) the dataset containing all complete neodermatan sequences and the incomplete *M. lineare *sequences as ingroups and four complete lophotrochozoan sequences as outgroups, (2) the dataset containing the complete genome sequences only (excluding *M. lineare *sequences), and using four lophotrochozoan sequences as outgroups, and (3) the dataset comprising five gene loci only (*nad5, cox3*, *atp6, cox1 *and *cob*) obtained universally from all platyhelminth ingroup and lophotrochozoan outgroup taxa. A multiple alignment for each gene loci was performed using ClustalX [51] with the following options: gap opening penalty = 10, gap extension penalty = 1.0 with a "delay divergent sequence" setting of 30% of the BLOSUM similarity matrix. The result of multiple sequence alignment is not always unambiguous due to the length and sequence variation among the taxa, causing the poorly aligned profile. Therefore, a conserved block of concatenated alignment was selected using the Gblocks program [52] for each of protein-coding loci of all species examined and then subjected to subsequent phylogenetic analyses. This was compared with an alignment where ambiguously aligned positions had been identified by eye; the two alignments were almost identical and yielded identical phylogenetic estimates. Molecular phylogenetic analyses of flatworm mitochondrial genomes were conducted using several methods applied to the protein sequence data. Bayesian analysis was performed using MrBayes 3.1 [53]. We set parameters to "ngammacat = 4", "rates = invgamma" for likelihood setting. Four Markov Chain Monte Carlo (MCMC) chains were run for 10^6 ^generations, sampled every 100 generations. Bayesian posterior probability values representing the percentage of samples recovering particular clades were estimated after initial 1,000 trees (the first 10^5 ^generations) were discarded. Maximum likelihood (ML) analysis was carried out using quartet puzzling method of the TREE-PUZZLE 5.2 program [54] under the mtREV24 matrix [55], as an evolution model for mitochondrial protein, with four categories of gamma-distributed rates estimated from the dataset. The analysis was run for 5×10^4 ^puzzling steps. For ML phylogenetic analyses, the mtREV substitution model of Adachi and Hasegawa was used as it is widely recognized to represent much better fit to the mtDNA-encoded protein sequence data than the Dayhoff and the JTT models [55,56]. The maximum likelihood mapping method [57] was conducted to assess the amount of phylogenetic signal in the dataset. We also conducted maximum parsimony (MP) analysis in PAUP* 4.0b10 version [58] and nodal support in the resulting tree was estimated by nonparametric bootstrap analysis with 1,000 random replications using a heuristic search option. Statistical tests for the alternative phylogenetic hypotheses were performed using the likelihood-based Shimodaira-Hasegawa test [41] and parsimony-based Templeton test [40] implemented in TREE-PUZZLE 5.2 and PAUP* 4.0b10, respectively.

## Authors' contributions

JKP designed this study, performed all of the phylogenetic analyses, interpreted data and wrote the manuscript. KHK carried out most of the molecular works. SK performed field survey for sampling the materials, assisted in the molecular work. WK and KSE provided phylogenetic and ecological insights. DTJL contributed with data analyses and writing the manuscript. All authors read and approved the final manuscript.

## Supplementary Material

Additional File 1 Circular representation of the mitochondrial genome of *Microcotyle sebastis*. All genes (not scaled) are encoded in the same direction and 22 tRNA genes (shadowed areas) are denoted by the one-letter code and two leucine and two serine tRNA genes are labeled, according to their anticodon sequence, as L1 (*trnL-uag*), L2 (*trnL-uaa*), S1 (*trnS-gcu*), and S2 (*trnS-uga*), respectively. The highly repetitive region (between *trnK *and UAR) and unassigned region (between HRR and *nad6*) are denoted as HRR and UAR, respectively. Click here for file

Additional File 2 Predicted secondary structures of the 22 mitochondrial tRNAs of *M. sebastis*. Click here for file
