# Supplementary figures and images for: A common origin of complex life cycles in parasitic flatworms: evidence from the complete mitochondrial genome of Microcotyle sebastis (Monogenea: Platyhelminthes)
Source: BMC Evol Biol. 2007 Feb 2;7:11. doi: 10.1186/1471-2148-7-11 (PMC1800851; doi:10.1186/1471-2148-7-11)

## Additional file 1

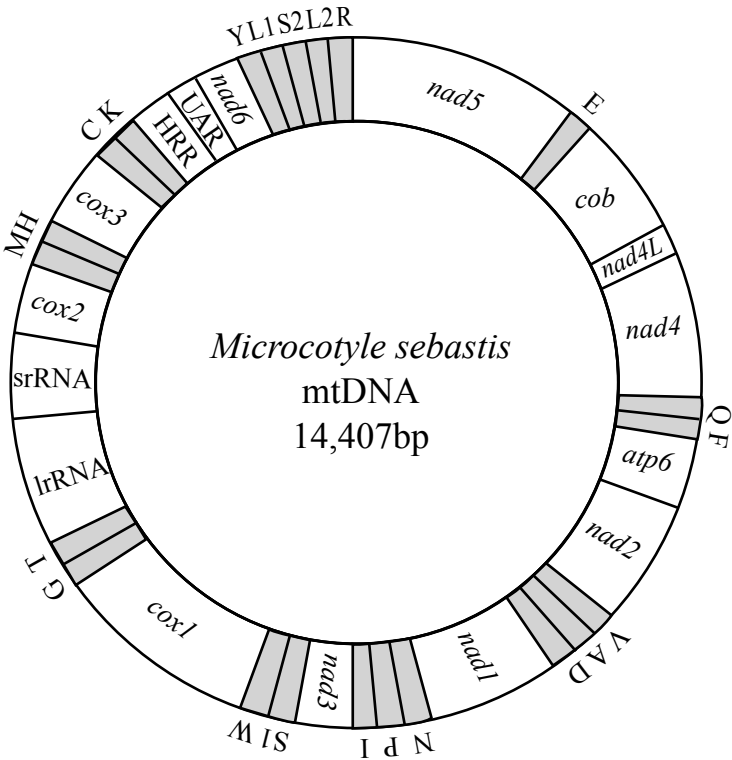

Supplement: Additional File 1 — Circular representation of the mitochondrial genome of Microcotyle sebastis. All genes (not scaled) are encoded in the same direction and 22 tRNA genes (shadowed areas) are denoted by the one-letter code and two leucine and two serine tRNA genes are labeled, according to their anticodon sequence, as L1 (trnL-uag), L2 (trnL-uaa), S1 (trnS-gcu), and S2 (trnS-uga), respectively. The highly repetitive region (between trnK and UAR) and unassigned region (between HRR and nad6) are denoted as HRR and UAR, respectively. [file 1471-2148-7-11-S1.pdf]

# Additional file 2

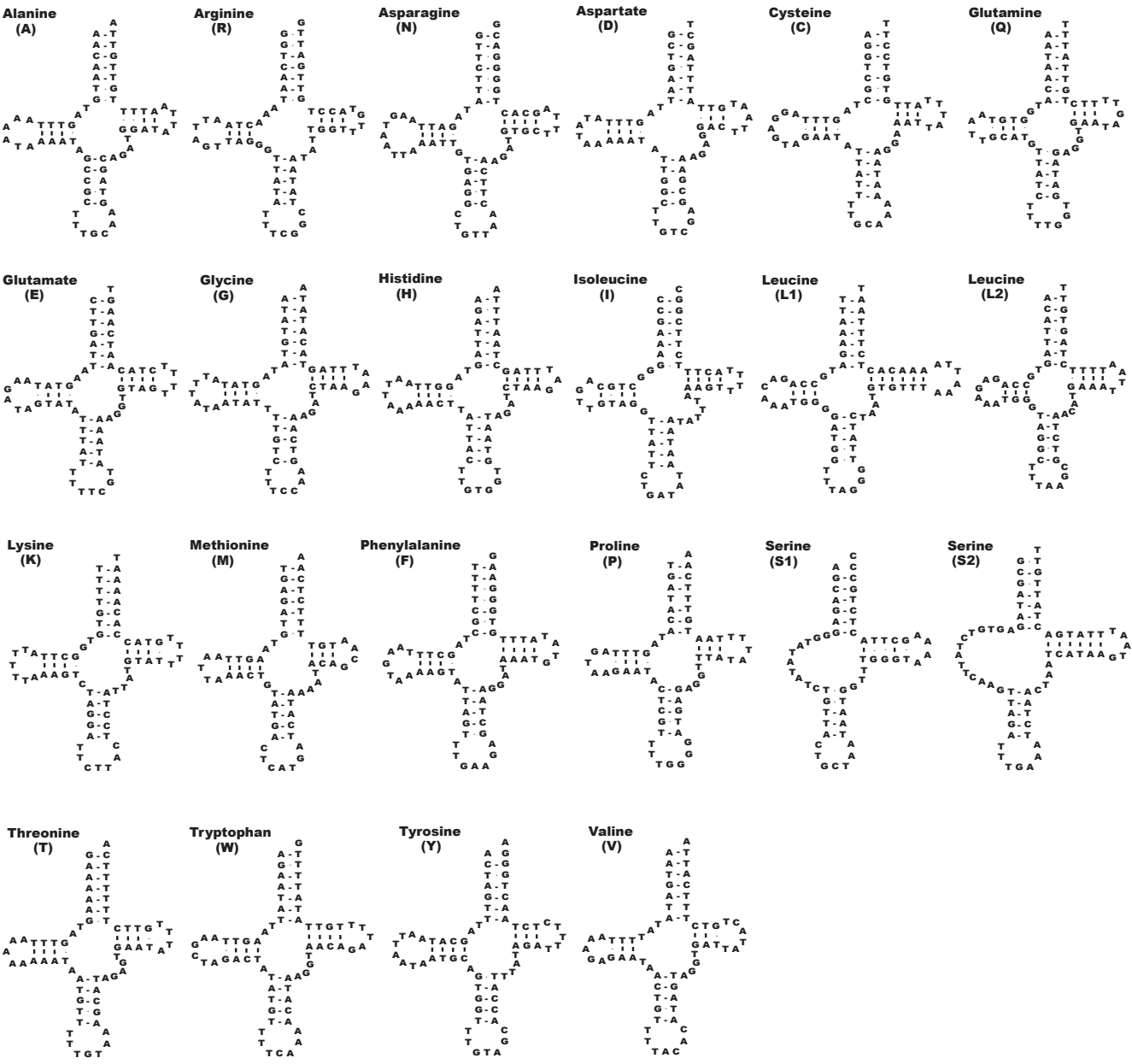

Supplement: Additional File 2 — Predicted secondary structures of the 22 mitochondrial tRNAs of M. sebastis. [file 1471-2148-7-11-S2.pdf]
